# Lack of consensus in the choice of termination of pregnancy for Turner syndrome in France

**DOI:** 10.1186/s12913-019-4833-3

**Published:** 2019-12-23

**Authors:** Monika Hermann, Babak Khoshnood, Olivia Anselem, Claire Bouvattier, Aurélie Coussement, Sophie Brisset, Alexandra Benachi, Vassilis Tsatsaris

**Affiliations:** 1 Service de Gynécologie-Obstétrique et Médecine de la Reproduction, Hôpital Antoine Béclère, Hôpitaux Universitaires Paris-Sud, Assistance-Publique Hôpitaux de Paris, Clamart, France; 2 Université Paris Sud, Le Kremlin-Bicêtre, France; 3 INSERM U1153 – Equipe de recherche en Epidémiologie Obstétricale, Périnatale et Pédiatrique (EPOPé) centre de Recherche Epidémiologie et Statistique Sorbonne Paris Cité (CRESS), Paris, France; 4 Maternité de Port Royal, Groupe Hospitalier Cochin-Broca-Hôtel Dieu, Assistance-Publique Hôpitaux de Paris, Paris, France; 5 Service d’endocrinologie pédiatrique, Hopital Bicêtre, Hôpitaux Universitaires Paris-Sud, Assistance-Publique Hôpitaux de Paris, Le Kremlin-Bicêtre, France; 6 Service de cytogénétique, Hôpital Cochin, Groupe Hospitalier Cochin-Broca-Hôtel Dieu, Assistance-Publique Hôpitaux de Paris, Paris, France; 7 Service de d’histologie, embryologie et cytogénétique, Hôpital Antoine Béclère, Hôpitaux Universitaires Paris-Sud, Assistance-Publique Hôpitaux de Paris, Clamart, France; 8 Université René Descartes, Paris, France

**Keywords:** Sex chromosome anomaly, Termination of pregnancy, Turner syndrome, Vignette study

## Abstract

**Background:**

The observed rate of termination of pregnancy (TOP) for Turner syndrome varies worldwide and even within countries. In this vignette study we quantified agreement among ten multidisciplinary prenatal diagnosis centers in Paris.

**Methods:**

We submitted online three cases of Turner syndrome (increased nuchal translucency, normal ultrasound, aortic coarctation) to fetal medicine experts: one obstetrician, one pediatrician and one geneticist in each of the ten Parisian centers. Each case was presented in the form of a progressive clinical history with conditional links dependent upon responses. The background to each case was provided, along with the medical history of the parents and the counseling they got from medical staff. The experts indicated online whether or not they would accept the parents’ request for TOP. We assessed the percentage of agreement for acceptance or refusal of TOP. We also used a multilevel logistic regression model to evaluate differences among obstetrician-gynecologists, pediatricians and cytogeneticists.

**Results:**

Overall agreement among the experts to accept or refuse TOP was, respectively, 25 and 28%. The percentage of disagreement was 47%. The percentage of agreement to accept TOP was 33, 8 and 33% for obstetrician-gynecologists, pediatricians and cytogeneticists, respectively. The respective percentages of agreement to refuse TOP were 19, 47 and 26%.

**Conclusion:**

Our results show the lack of consensus with regard to decisions related to termination of pregnancy for Turner Syndrome. This lack of consensus in turn underscores the importance of multidisciplinary management of these pregnancies in specialized fetal medicine centers.

## Background

Turner syndrome affects 1/2500 of female newborns [1], ie, approximately 1/5000 of all live births. Most fetuses with a 45,X karyotype die in utero and it is estimated that only 1% of fetuses with monosomy X are viable, whence the relatively low prevalence of Turner syndrome [1]. Turner syndrome is caused by complete or partial monosomy for the X chromosome: half of the patients have a 45,X karyotype; the remainder are mosaics with a 45,X cell line and some have a structurally abnormal X (ring X chromosome). Phenotypic expression is variable and depends on the chromosomal formula. Women with mosaicism generally have less severe impairment and an excellent prognosis regarding intelligence, whereas women with ring X chromosome have a guarded prognosis concerning cognitive impairment. Growth restriction is almost always present and results in an adult stature 20 cm below the reference population mean [2, 3], but is partially ameliorated by growth hormone therapy [4–7]. Intellectual ability may depend on hearing impairment, which ranges from hypoacusis to deafness [8–10]. Overall, cognitive deficits are seen in 6 to 10% of patients, who need special schooling [11]. Quality of life during adolescence depends on the burden of medical follow-up (in the case of cardiopathy, auto-immune disease, etc) and induction of puberty [12–16]. Infertility is almost always present. Quality of life and long-term prognosis depend on the associated conditions [11, 17, 18]: cardiopathy (aortic coarctation in 5 to 10% of cases, bicuspid aortic valves in 15%), hypothyroidism in 30% of cases [19, 20], renal anomalies (30–40%) [16]. Long term, Turner syndrome patients have higher morbidity and mortality than the general population because they are at greater risk of hypertension, obesity and osteoporosis [21–23].

Turner syndrome can be diagnosed in utero from the first trimester when there is an increased nuchal translucency or lymphedema (Bonnevie-Ullrich syndrome) or later when there is a prenatal warning sign. Its prenatal discovery may also be fortuitous when karyotyping is done for other reasons, usually when first-trimester serum marker levels indicate increased risk of trisomy 21. Prevalence of prenatal detection of Turner syndrome is low and variable over time – from 1/300 in the 1980’s to around 30% nowadays [1, 24]. The advent of noninvasive prenatal diagnosis, the indications of which are increasingly wide, also increases the frequency of fortuitous detection of gonosomal abnormalities.

Elective TOP is performed in 54 to 100% of cases of Turner syndrome in Europe and North America, despite the low risk of intellectual deficit and absence of mental retardation [25–28]. Legislation on TOP differs among countries in terms of the number and qualification of specialists in charge with affirming the serious and incurable nature of the fetal disease, the maximum term at which TOP can be performed, and the legal time for reflection between when the parents are told of fetal disease and TOP [29]. In France, fetal diseases are managed in multidisciplinary prenatal diagnosis centers (PDCs), which help medical teams and parents in analysis, decision making, and pregnancy follow-up when a malformation or fetal anomaly is suspected and when there is a genetical transmission of a disease (article 2131–10 of the Public Health Code). When there is a high probability that the unborn child has a particularly severe condition deemed incurable at the time of diagnosis, the role of the PDCs is to certify this (article 2231–1 of the Public Health Code). This opens the way, should the parents wish, to TOP for medical reasons. Unlike other European countries, in France there is no time limit on when TOP can be performed. There is a mandatory 7-day period for parents to think over their decision between diagnosis of a severe disease and TOP. The severe and incurable nature of the fetal disease must be certified by two specialists of a PDC: if possible, one specialist of the disease the fetus appears to have and one obstetrics specialist (article L.2213–1 of the Public Health Code) [30]. Access to elective TOP for Turner syndrome is subject to numerous ethical tensions. In the EUROCAT database [28, 31], which collects data on congenital malformations in Europe, the rate of elective TOP for gonosomal abnormalities varies considerably among countries, even when the laws governing TOP are similar [25]. There is also variability among PDCs within a given country [26, 32]. Nevertheless, reasons for this observed variability are unclear and literature has not yet been explored if this difference is due to the profession of the * PCDs staff members, to local laws upon TOP or severity of antenatal signs. Neither has the variability of the decisions been quantified with precision regards to the decision for TOP. Overall rates of TOP hence compare heterogeneous prenatal situations. Indeed, studies compare overall rates of TOP for Turner syndrome [25, 27, 28]: they do not distinguish which signs led to prenatal diagnosis. Both physicians implicated in the decision-making process and patients have different characteristics. Quantification and knowledge of the reasons in different approaches among PDCs could influence their organization, including the way the information is given to patients’ and decisions to perform invasive procedures.

Our aim was therefore to study the agreement to accept or not parents’ demand of TOP for Turner syndrome among:

- several PDCs in the Paris–Île de France region in standardized clinical cases presented in the form of a vignette.
- different professionals working in a PDC.

## Methods

**Fig. 1:**
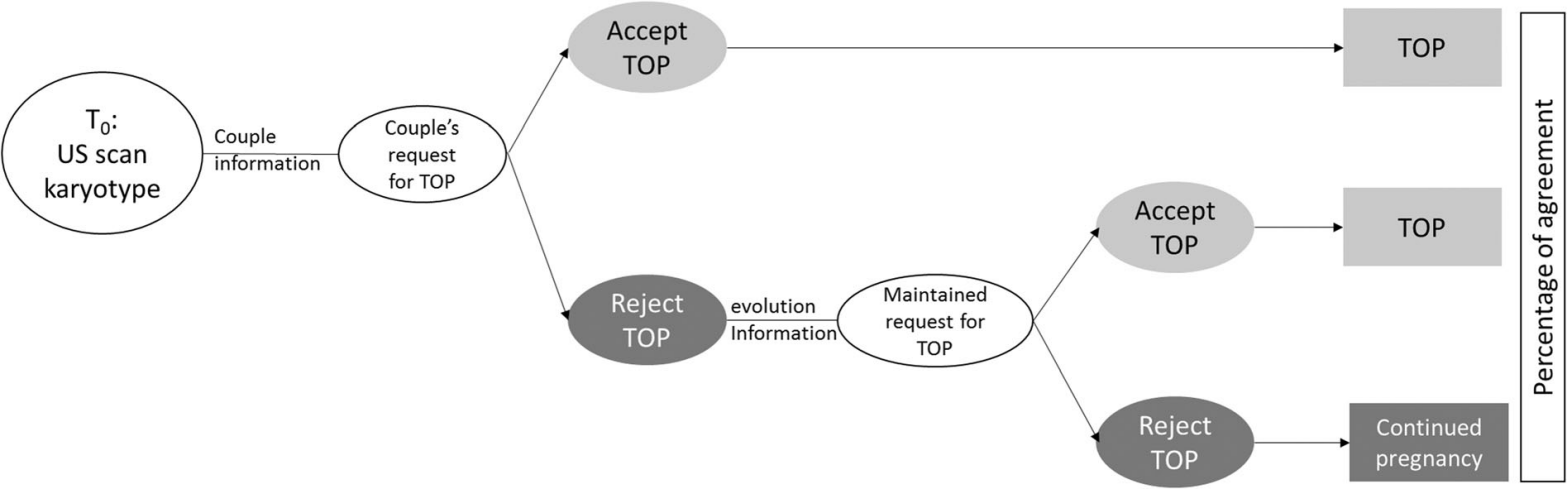
Online presentation of clinical cases (vignettes). TOP: termination of pregnancy

We conducted a survey called DAGO (antenatal diagnosis of gonosomal abnormalities) of practices in PDCs in the Paris–Île de France region. Three online vignettes derived from real-life clinical cases were prepared and approved by a scientific committee composed of four obstetrician-gynecologists (AB, OA, MH, VT), an endocrinologist-pediatrician (CB) and two cytogeneticists (AC, AC): Turner syndrome with nuchal translucency of 7 mm in the first trimester; Turner syndrome with no ultrasound sign (discovered on amniocentesis done for an increased risk of Down syndrome estimated on first trimester markers); Turner syndrome discovered in the third trimester because of aortic coarctation. Each case was presented in the form of a progressive clinical history with conditional links dependent upon responses. The background to each case was provided, along with the medical history of the parents, the reason for antenatal diagnosis, the invasive examination that led to karyotyping, the result of the karyotyping, the information given to the parents, and ultrasound findings before and after the invasive examination. Psychological follow-up was routinely proposed to parents, who saw a pediatric endocrinologist before taking any decision regarding the pregnancy. The information on the prognosis given to the parents was that on the patient information sheet available online at Orphanet (http://www.orpha.net/). After receiving multidisciplinary information, parents decided to formulate a request for elective TOP, and experts were then asked whether or not they would grant this request (cf Fig. 1).

Depending on the online respondent’s answer, the experts who would accept TOP were directed to the next clinical case, whereas the experts who refused TOP continued with the same clinical case. A report on clinical progression indicated the psychological progression of the parents, the ultrasound findings and how they changed, appointments with other specialists, or new appointments with the pediatric endocrinologist. The parents then requested TOP again, despite the proposed medical and psychological support. We then asked the experts who had initially refused TOP whether they would have accepted it in this context. We present the percentages of agreement regarding the final decision (including the experts who would have accepted TOP at the outset and those who would have accepted it in the light of clinical progression), as this is the only figure that is comparable with the literature data and real-life situations.

We submitted the three online clinical cases using www.evalandgo.com to 30 prenatal diagnosis experts in the 10 PDCs in the Paris-Île de France region. The contacted members received a link to answer the survey. The vignettes were presented as clinical cases. Questions were asked progressively and depending on the answers. If the person answering the questionnaire agreed with TOP in the first place, he would be directed to the next vignette. If he answered he refused TOP, the clinical case was continued.

In each PDC, we asked an attending obstetrician-gynecologist, an attending pediatrician and a cytogeneticist or geneticist. Other professionals work in these PDCs, and the center’s final decisions includes the opinions of all participants, including midwives, fetal pathologists, obstetrician-gynecologists other than the attending obstetrician-gynecologist, pediatricians other than the attending pediatrician, etc. To optimize recording of the decisions taken by the PDCs, we decided to question members exhaustively in two centers. So, we submitted the same three clinical cases to all staff members in these two PDCs, including all obstetrician-gynecologists, pediatricians, geneticists, cytogeneticists, midwives, sonographers and fetal pathologists.

For each clinical case, we calculated the percentage of agreement to accept the request for TOP, the percentage of agreement to refuse TOP and the percentage of disagreement. We did so using the method initially described by Chamberlain [33] and later generalized by Grant [34] to tests for more than two observers.

We asked 30 experts. We obtained 27 fulfilled questionnaires. We calculated the agreement among observers in favor of accepting a request for TOP as e = n_i_ (n_i_– 1)/2 where ni was the number of experts who thought TOP was justified and thus said “yes” to TOP in the online questionnaire. We calculated agreement to refuse a request for TOP as g = m_i_ (m_i_– 1)/2 where mi was the number of experts who thought that a TOP was not justified. Then we calculated the sum of agreement to accept TOP for all the vignettes as $\mathsf{C}\mathsf{ifor}={\sum}_3^1e$ and the sum of agreement to refuse TOP as ${C}_{iagainst}={\sum}_3^1g$. Disagreement concerning a decision for TOP was alculated as $f=\frac{\left(\left({n}_i+{m}_i\right)\ast \left({n}_i+{m}_i-1\right)\right)}{2}-e-g$. Sum of disagreement was calculated as ${D}_i={\sum}_3^1f$ (Additional file 1: Table S1). Percentage of agreement to accept a request for TOP was calculated as ${P}_{accept}=\frac{C_{ifor}}{\left({C}_{iagainst}+{C}_{ifor}+{D}_i\right)}$. Percentage of agreement to refuse a request for TOP was calculated as ${P}_{refuse}=\frac{C_{iagainst}}{\left({C}_{iagainst}+{C}_{ifor}+{D}_i\right)}$.

Percentage of disagreement was calculated as ${P}_{disagree}=\frac{D_i}{\left({C}_{iagainst}+{C}_{ifor}+{D}_i\right)}$.

We also used a multi-level (hierarchical) logistic regression analysis in order to test the possible effect of specialty on decisions of experts to accept (or not) a request for TOP. We used a multi-level model in order to take into account the hierarchical cases (vignettes) nested within experts (of different specialties), in turn nested within centers.

For the agreement analyses we used Excel (Microsoft Office, version 15.0.4737.1003). For the multilevel model, we used the multilevel logistic model (melogit) of Stata software (Stata 13.1, Texas, USA).

This study was approved by the Ethical Review Committee « Comité d’éthique de la recherche en obstétrique et gynécologie » under the number CEROG OBS 2017-02-26. This research was found to conform to generally accepted scientific principles and medical research ethical standards by the upper stated institutional review board.

## Results

**Fig. 2:**
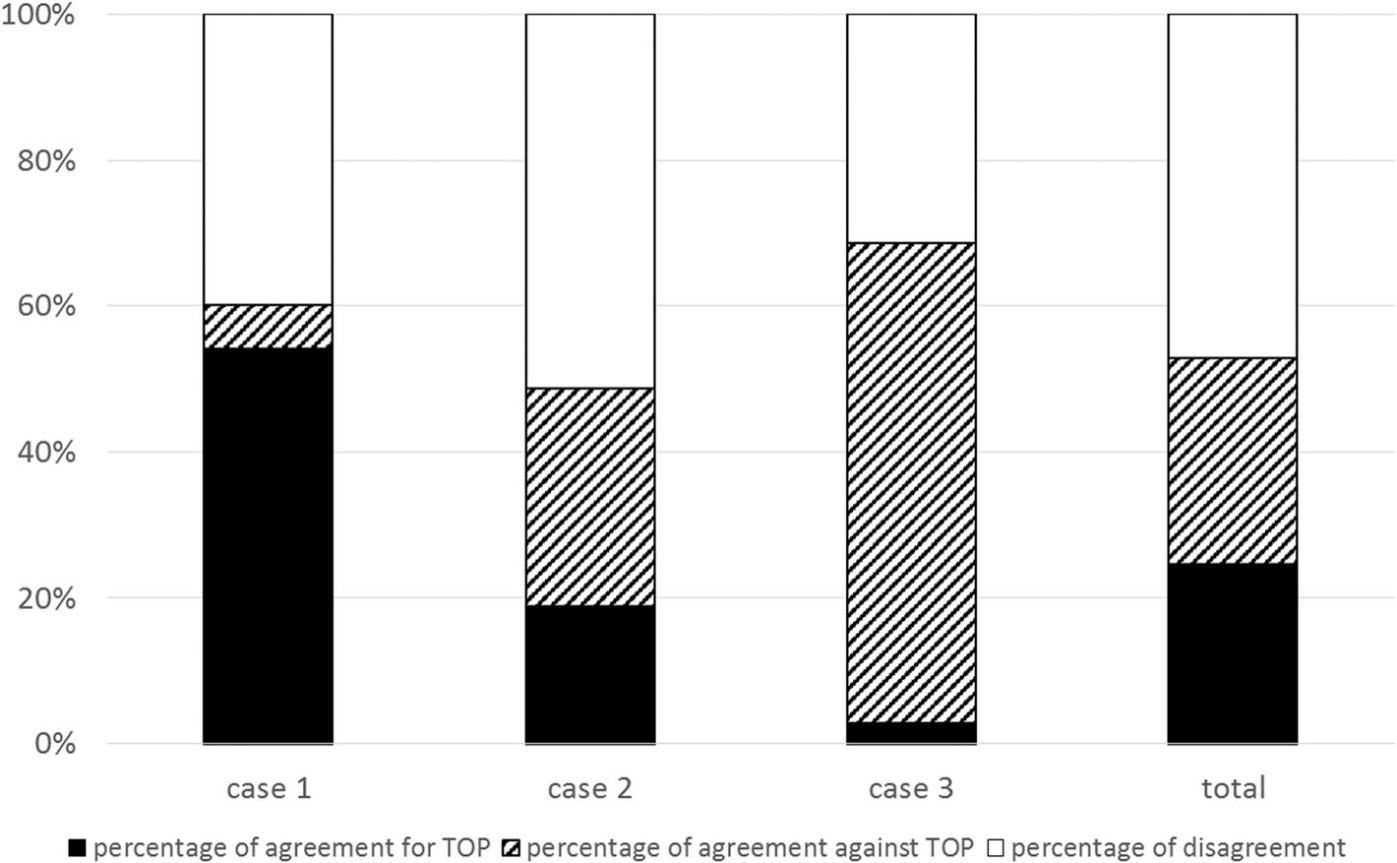
Percentage of agreement to accept a request for termination of pregnancy (TOP), agreement to refuse a request for TOP and disagreement in the 10 prenatal diagnosis centers

We received 27 responses from the 30 experts in the 10 PDCs. For the first clinical case (discovery via nuchal translucency), the percentage of agreement to accept TOP was 54% and the percentage of agreement to refuse TOP was 6%. For the second clinical case (serendipitous discovery), the percentage of agreement to accept and to refuse TOP were respectively, 19 and 30%. For the third case (discovery because of aortic coarctation), the respective percentages were 3 and 66% (Fig. 2). For the three clinical cases taken together, the percentage of agreement to accept TOP was 25%, the percentage of agreement to refuse 28% and the percentage of disagreement was 47% (Fig. 2).

**Fig. 3:**
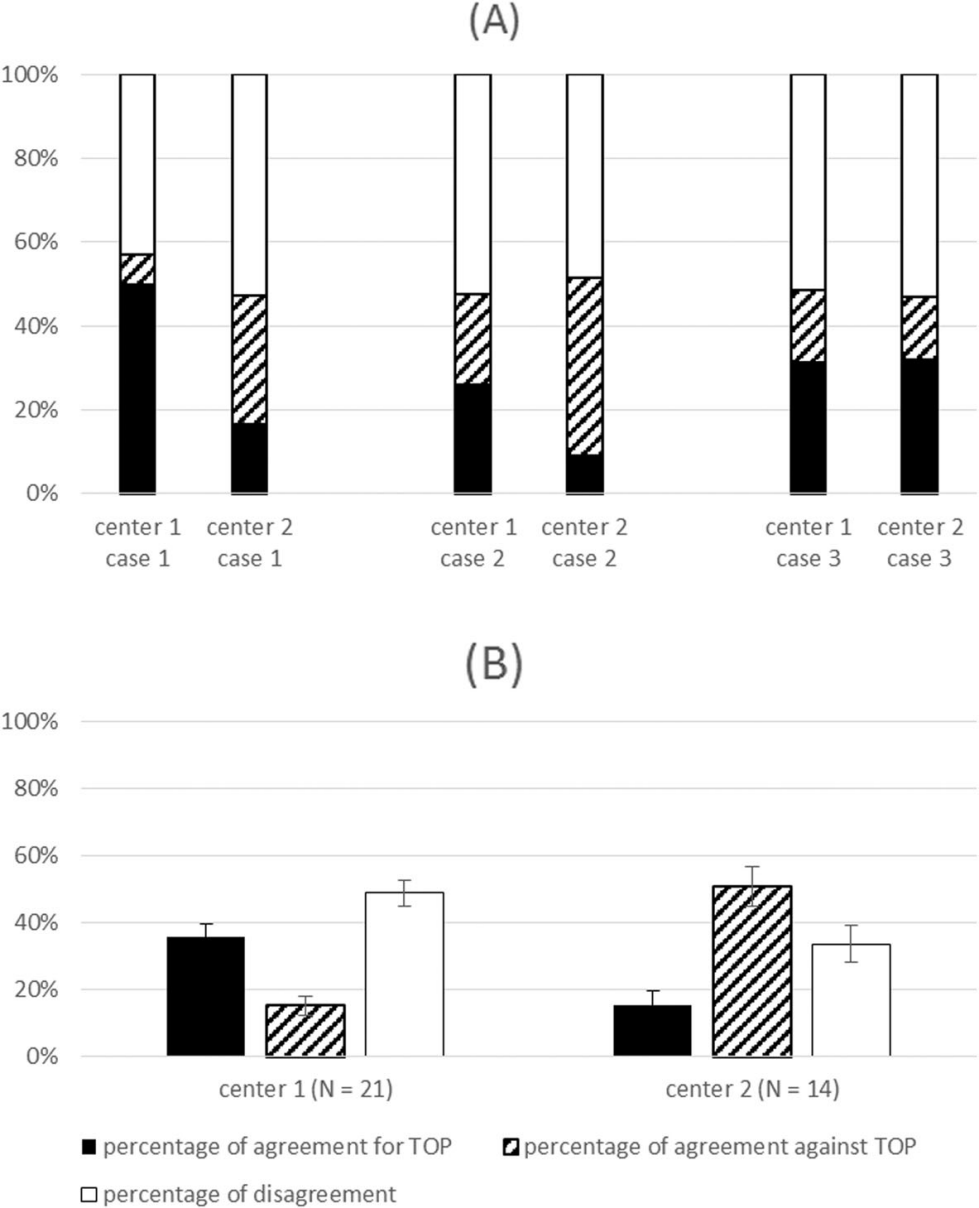
Percentage of agreement to accept TOP, agreement to refuse TOP and disagreement in centers 1 and 2. **a** Percentages case by case. **b** Overall percentages for the three cases. The error bars represent the 95% confidence intervals. Black: percentage of agreement to accept TOP. Diagonal lines: percentage of agreement to refuse TOP. White: percentage of disagreement

In the two centers where we questioned all staff members, we obtained 21 responses in center 1 (9 gynecologist-obstetricians, 4 pediatricians, 2 sonographers, 1 midwife, 1 anatomic pathologist, 1 geneticist and 2 cytogeneticists) and 14 responses in center 2 (6 gynecologist-obstetricians, 3 pediatricians, 2 sonographers and 3 cytogeneticists). The percentage of agreement to accept TOP was for cases 1, 2 and 3, respectively, 50, 26 and 31% in center 1 and 16, 7 and 32% in center 2. The overall results for the 3 cases are shown in Fig. 3.

**Fig. 4:**
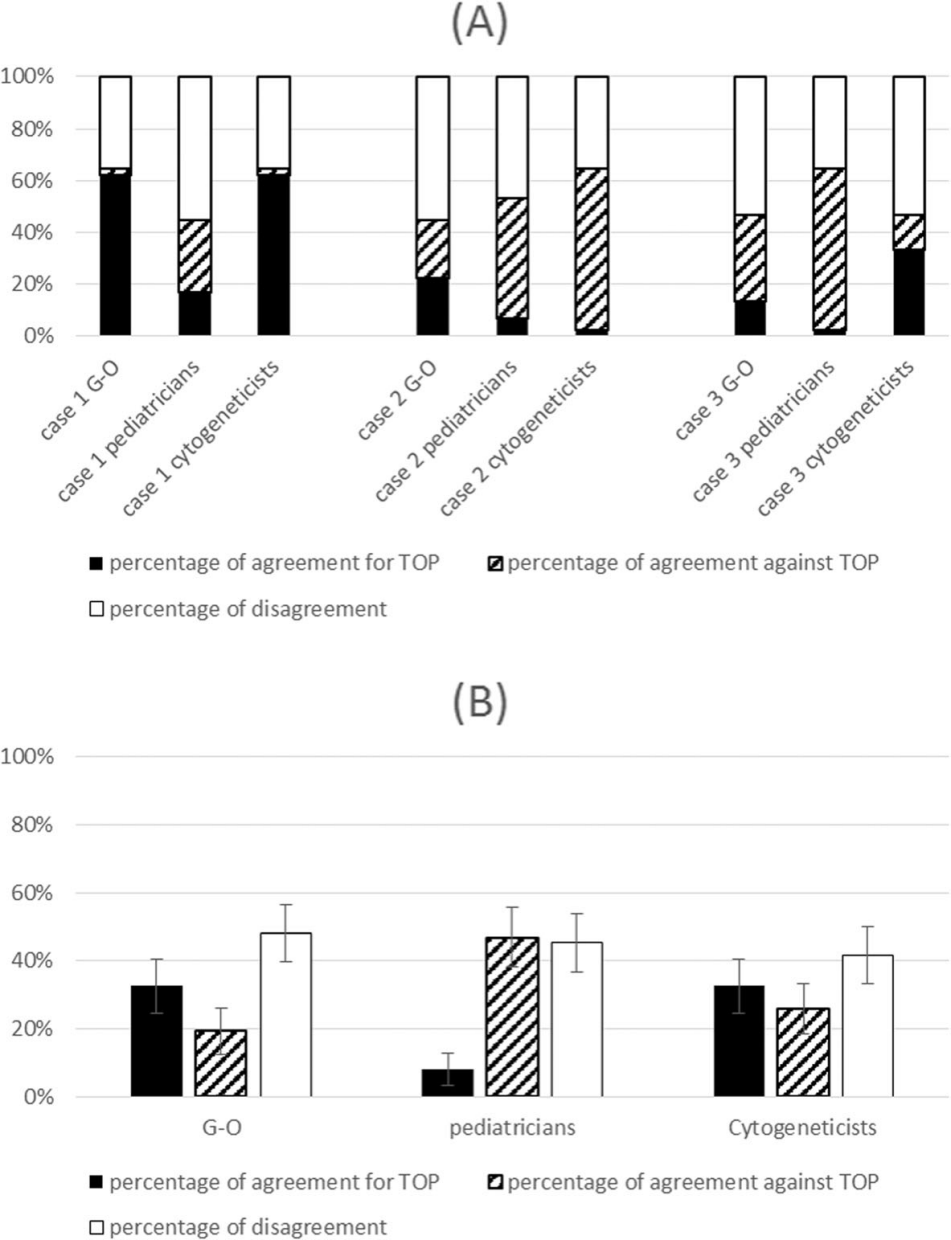
Percentage of agreement to accept TOP, agreement to refuse TOP and disagreement by profession (obstetrician, pediatrician, cytogeneticist). **a** Percentages case by case **b** Overall percentages for the three cases. G-O: gynecologist-obstetricians

In Fig. 4, we show results within each of the three specialties. The overall percentages of agreement (for the three cases together) to accept TOP were, respectively, 33, 8 and 33% for gynecologist-obstetricians, pediatricians and cytogeneticists (Fig. 4). The percentage of agreement to refuse TOP was, respectively, 19, 47 and 26% (Fig. 4).

Our multilevel model shows that the variance of the center was 0.32 (95% CI: 0.06–1.76) and that of the experts 0.05 (95% CI 0.000001–2205). There was a significant effect of the center (level 3) and of the experts (level 2).

The results of the multilevel, multivariate analysis for the final decision to approve TOP are presented in Table 1. There was no significant difference among specialties in univariate or in multivariate analysis.

**Table 1:** Multivariate analysis of the probability of agreeing to elective TOP or not

Final decision to approve TOP	ORa^a^ (95% CI)
Gynecologist-obstetricians	Reference
Pediatricians	0.51 (0.21–1.23)
Cytogeneticists/geneticists	0.59 (0.27–1.27)

^a^Odds ratio adjusted for the syndrome and the specialty in a multilevel logistic regression (level 1: the decision, level 2: the experts, level 3: the PDC)

## Discussion

Prenatal counseling after diagnosis of Turner syndrome is complex. The risk of intellectual disability is low (6 to 10%), but may seem unacceptable to some parents. Other issues like infertility and long-term medical complications lead parents to request TOP following sometimes serendipitous prenatal discovery of Turner syndrome. However, the members of PDCs may not have the same perception of these postnatal complications and may not accept this request. Overall, we found lack of consensus regarding choice of TOP for Turner syndrome; this was reflected in both the percentages of disagreement among experts and in the low percentages of agreement to accept or to refuse TOP. Our results show differences in the experts’ decisions depending on the mode of discovery of Turner syndrome and associated ultrasound findings: higher percentages of agreement were found when Turner syndrome was diagnosed on increased nuchal translucency, whereas higher percentages of disagreement were found when Turner syndrome diagnosis was serendipitous. Morover, variations can also possibly be explained by the gestational age of diagnosis of Turner syndrome. Indeed, as shown Fig. 2, higher percentage of agreement against TOP were found for case 3, when Turner syndrome was diagnosed during the third trimester of pregnancy. Finally, there appeared to be a specialty effect with pediatricians accepting less often a TOP request even if most likely due to low power this association was not statistically significant. Indeed, this study lacks of sample size calculation. We send the survey to all the Prenatal Diagnosis Centers in the Parisian region. We contacted one obstetrician, one pediatricien and one genetician in each center. All except three ansered to the survey.

**Fig. 5:**
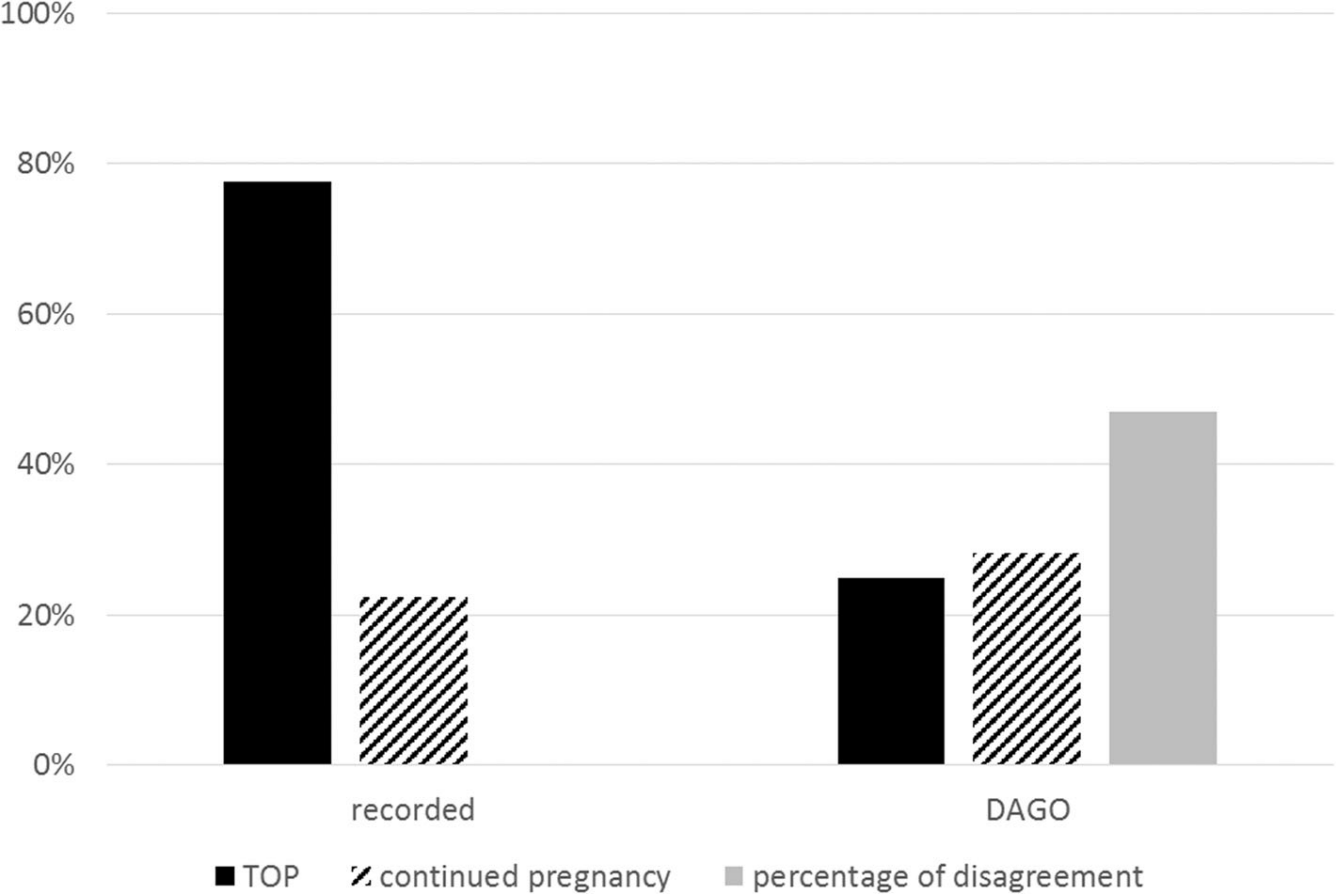
Comparison between our study data (DAGO) and the Paris Registry of Congenital Malformations 2008–2012

The stance of the experts in this study seems to differ from the situation in real life, where approximately 78% of pregnancies are terminated when Turner syndrome is diagnosed, according to the Paris Registry of Congenital Malformations [35]. In our study there was 72% agreement to accept TOP, which corresponds to only 48% of TOP which would have finally been accepted (cf Figs. 2 and 5). There are several possible explanations for the mismatch between our results and real-life findings. First, our study differs from what is seen in practice. Real-life follow-up may include other aspects, notably ultrasound signs not found in our clinical cases. Such signs can also contribute to acceptance of TOP, particularly if they worsen the prognosis. Also, having met the parents, the experts will be particularly sensitive to their request. Their position will be influenced by the particular situation of the parents, whereas other members of the PDC will be more neutral and will discuss the indication for TOP on a theoretical basis. Experts’ decisions in this survey were not influenced by the pressure usually made by couples requesting TOP and their caring practitioners. Indeed, the doctors who met the parents usually play an important role in the final decision-making within the PDC. Morover, variations in the population served in different PDCs could influence decisions of some PDCs beyond the law. Cultural and ethical differences influence the number of requests for TOP and hence the caregivers might be more willing to accept TOP within populations who request it more rarely.

In fact, the sum of the percentage of agreement to approve TOP and the percentage of disagreement were highly comparable to the observed percentage of TOP in registries (cf Fig. 5). The decision by PDC members to accept TOP for Turner syndrome is therefore not necessarily unanimous. It is most probable that a TOP is accepted within PDCs either if members of a PDC unanimously accept the parents’ request for TOP, or either if some members accept it. Indeed, the last situation is in accordance with the law in France as long as two specialist members of the PDC recognize the incurability and seriousness of the case [30]. The structure of the PDCs is thus designed to guarantee the best possible match between the parents’ request and the law, by accompanying the PDC’s decision with an interpretation of the individual perception. This over-representation of TOP for Turner syndrome in registries may also be related to how information was first given to the parents. The initial information has a major impact on the parents’ final decision. Sometimes the parents are informed of the results of fetal karyotyping by a health care worker who does not really know the prognosis of Turner syndrome. In this regard it is essential to optimize in each PDC how and by whom the parents are told of the karyotyping result. The announcement should be made by the specialist in the disease and its prognosis, and a consultation with an endocrinologist-pediatrician and a cytogeneticist should be arranged without delay.

We do not think it will be possible to clarify guidelines for prenatal prognosis of Turner syndrome as it will always represent a broad phenotypical spectrum. Neither do we hope to have a limited list of fetal pathologies for which TOP seems to be acceptable. Nevertheless, serendipitous diagnosis of Turner syndrome will probably be more frequent with the growing use of non invasive prenatal diagnosis. Hence the results of this study insist on PDCs’ role to avoid a drift of decisions towards inadequate or hasty decisions of TOP especially in a country were TOP is allowed at any gestational age.

## Conclusions

We found a low percentage of agreement for TOP for Turner syndrome, and lower than what is observed in real life. Differences among members of PDCs are highlighted by high percentages of disagreement. This study illustrates the complexity attendant upon decisions relating to termination of pregnancy in cases of Turner syndrome. It witnesses the ethical tensions linked with these decisions; in particular for Turner’s syndrome without anomalies on the obstetrical ultrasound scans. The multidisciplinary organization of prenatal diagnosis centers is determinant in meeting requests for termination of pregnancy, considering both the disease and the parents’ characteristics. The knowledge of these differences might influence the organisation within PDC related to the way the information is given to parents concerning invasive and noninvasive procedures which could lead to a serendipitous discovery of the anomaly.

## Supplementary information

**Additional file 1: Table S1.** Table generalizing Grant’s method for more than two observers.

## Data Availability

The datasets supporting the conclusions of this article are available upon request to the corresponding author.

## Abbreviations

DAGO: Antenatal Diagnosis of Gonosomal Abnormalities
PDC: Prenatal Diagnosis Center
TOP: Termination of Pregnancy
