# Supplementary material for: Lack of consensus in the choice of termination of pregnancy for Turner syndrome in France
Source: BMC Health Serv Res. 2019 Dec 23;19:994. doi: 10.1186/s12913-019-4833-3 (PMC6929477; doi:10.1186/s12913-019-4833-3)
Supplement: Supplementary file 1 — Additional file 1: Table S1. Table generalizing Grant’s method for more than two observers. [file 12913_2019_4833_MOESM1_ESM.docx]

**SUPPLEMENTAL MATERIAL**

**Table S1. Table generalizing Grant’s method for more than two observers.**

| **Vignette** | **TOP accepted** | **Agreement to accept TOP** | **Disagreement** | **Agreement to refuse TOP** | **TOP refused** |
| --- | --- | --- | --- | --- | --- |
| **1** | 20 | 190 | 140 | 21 | 7 |
| **2** | … | … | … | … | … |
| **… i …** | n_i_ | $e$ = n_i_ (n_i_– 1)/2 | $f$ $=\frac{(\left( n_{i}+ m_{i} \right)*\left( n_{i}+ m_{i}-1 \right))}{2}-e -g$ | $g$ = m_i_ (m_i_– 1)/2 | m_i_ |
| **Total** |  | $\text{C}\text{ifor}=\sum_{3}^{1} e$ | $D_{i}=\sum_{3}^{1} f$ | $C_{iagainst}=\sum_{3}^{1} g$ |  |
